# The role of GSTM1 gene polymorphisms in lung cancer development in Turkish population

**DOI:** 10.1186/1477-3163-6-13

**Published:** 2007-09-26

**Authors:** Adalet Demir, Sedat Altin, Davut Pehlivan, Mulahim Demir, Fatih Yakar, Ekrem Cengiz Seyhan, Seyyit Ibrahim Dincer

**Affiliations:** 1 Yedikule Teaching Hospital for Chest Diseases and Thoracic Surgery, Depertmant of Thoracic Surgery, Istanbul, Turkey; 2 Istanbul University, Istanbul Medical Faculty, Department of Medical Genetics, Istanbul, Turkey; 3 Istanbul University, Istanbul Medical Faculty, Department of Chest Diseases, Istanbul, Turkey

## Abstract

**Background:**

Glutathione S-transferase (GSTs) plays an important role in the detoxification of many xenobiotics involved in the etiology of cancer. In different ethnic groups, variations in null allele frequency have been observed. We have investigated GSTM1 gene polymorphisms in healthy subjects and lung cancer patients in the Turkish population and reviewed the control subjects of the studies performed in the Turkish population.

**Methods:**

Following blood sampling from patients and controls, DNA samples were extracted from the whole blood and were amplified by using polymerase chain reaction (PCR) method in all of the 256 cases, consisting of 102 previously diagnosed with lung cancer and 154 healthy controls.

**Results:**

The prevalence of GSTM1-null genotype in the lung cancer patients was 49%, compared to 52.6% in the control group (OR = 1.39, 95% CI = 0.70–1.90, p = 0.57). There were also no significant relationships in GSTM1 genotypes among histopathologic types of lung cancers (p > 0.05). The frequency of GSTM1 was found to be 41.2% (n = 1809) when the control subjects of the studies performed in Turkish population were reviewed.

**Conclusion:**

We have observed that GSTM1 genotype is not an independent risk factor for lung cancer.

## 1. Background

Carcinoma of the lung is the most common cancer and the most frequent cause of death in the patients with cancer around the world [1]. Environmental carcinogens such as active and passive smoking, air pollution and environmental exposures have strong influences on individual factors [2]. In humans, there are several genetic polymorphisms of the enzymes involved in metabolic activation and detoxification of pulmonary carcinogens including polycyclic aromatic hydrocarbons (PAH) and aromatic amines. Interindividual differences in ability to activate and detoxify carcinogens are expected to affect the risk of developing lung cancer [3]. Polymorphisms of the genes encoding phase I and phase II xenobiotic metabolizing enzymes have been shown to be associated with susceptibility to lung cancer in a number of epidemiologic studies [4]. However, most of these studies are limited by lack of adequate statistical power. To overcome this limitation, the International Collaborative Study on Genetic Susceptibility to Environmental Carcinogens (GSEC) has begun and is on-going to pool raw data of studies on metabolic genetic polymorphisms and cancer risk [5].

Glutathione S-transferase (GSTs) plays an important role in cellular defense mechanism since they are involved in detoxification of many carcinogens and environmental pollutants and facilitate their excretion and also have a role in protection against oxidative stress [6,7]. The frequencies of polymorphic genes in control populations have been reported to be different in various ethnic groups. In addition, interethnic differences have been established [7-9]. GSTM1 deletion frequencies range from 42% to 60% in Caucasians [8].

Some studies suggest that the GSTM1 null genotype confers an increased risk of lung cancer but this result has not been approved by others, especially recent meta and pooled analysis [5,7,10-13].

The aims of the present study are to evaluate the frequencies of GSTM1 gene polymorphisms in Turkish population and whether genetic polymorphisms in GSTM1 influence individual susceptibility to lung cancer in Turkish population or not.

## 2. Methods

### 2.1. Study subjects and sample collection

A total 256 subjects, composed of 102 lung cancer patients, who were admitted to Yedikule Teaching Hospital for Chest Diseases and Thoracic Surgery in Istanbul between 2001–2005, and 154 healthy controls were included in this study. All cancer patients and controls were born in Turkey. The control group had neither cancers nor chronic diseases. The mean ages were found to be 56.3 ± 10 (range 30–75) and 35.1 ± 11 (range 20–65) in cancer group and healthy controls, respectively. Ninety-four patients in cancer group and 110 subjects in healthy controls were smokers. This study was approved by local hospital ethics committee on human research. All patients gave informed consent.

### 2.2. GSTM1 genotyping

**Figure 1:**
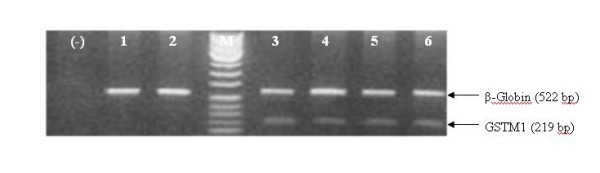
GSTM1 genotyping. (-): water; 1, 2 : GSTM1 null genotype (0/0); 3, 4, 5 and 6 GSTM1 +/+ or +/- genotype. M: 100 bp ladder size marker.

DNA samples were amplified with the primers: 5'- GAACTCCCTGAAAAGCTAAAGC -3' and 5'-GTTGGGCTCAAATATACGGTGG-3' for GSTM1 which produced a 219 bp product [14]. The PCR amplification was carried out 1 μg DNA in 10 mM Tris-HCl, pH 8.3, 50 mM KCl, 3 mM MgCl2, 0,3 mM deoxyribonucleotide triphosphates (Fermentas), 0,2 μM of each primer and 1,5 U of Taq polymerase (Fermentas) in a total volume of 50 μl. Amplification was performed with initial denaturation at 94°C for 5 minutes, followed by 30 cycles at 94°C for 1 minute, 61°C for 1 minute, and 72°C for 1 minute, and a final extension at 72°C for 10 minute, using a MJ Research PTC160 thermal cycler. The amplification product (10 μl) was visualized in an ethidium bromide stained 1.5% agarose gel. All the genotype determination were carried out twice in independent experiments and all the inconclusive samples were reanalyzed. The results are shown in figure 1.

### 2.3. Statistical analysis

Statistical analyses were performed using the Statistical Package for the Social Sciences Program (SPSS, Version 10). Pearson's X^2 ^test was used to examinutee differences with regard to demographic variables, smoking and distribution of genotypes. Associations between the GSTM1 polymorphisms and risk of lung cancer were estimated using odds ratios (ORs) and 95% confidence intervals (95% CIs) calculated by conditional logistic regression.

## 3. Results

The demographic characteristics of the cancer group and healthy controls are shown in the Table 1. The prevalence of GSTM1 (0/0) genotype in the cancer group was 49% compared to 52.6% in control group. But the difference was not statistically significant (OR = 1.39, 95% CI = 0.70–1.90, P = 0.55) (Table 2).

**Table 1:** Demographic characteristics of the lung cancer patients and controls

Characteristics	Patients (n = 102)	Controls (n = 154)
Mean	56.3 ± 10	35.1 ± 11
Range	(30–75)	(20–65)
Male	91	98
Female	11	56
Smokers	94	110
Non-smokers	8	44
Cigarettes (*Package/year*)	39.2 ± 4.2	14.5 ± 3.2
Histopathology		
Epidermoid carcinoma	64(62.7%)	
Adenocarcinoma	25(24.5%)	
Others	13(12.7%)	

**Table 2:** Frequency of GSTM1 genotypes in lung cancer patients and controls

Genotypes	Patients n (%)	Control n (%)	OR(95%) CI	p value
GSTT1				
Present	52(51%)	73(47.4%)	1.0	
Null	50(49%)	81(52.6%)	1.39(0.70–1.90)	0.57

*Abbreviations*: OR, odds ratio; CI, confidence interval

Histopathological evaluation, performed according to WHO criteria, revealed that epidermoid carcinoma, adenocarcinoma and the others types were present in 62.7% (n = 64), 24.5%(n = 25) and 12.7% (n = 13) out of 102 cancer group, respectively. There was no statistically significant difference among the histopathologic types of lung cancer (p > 0.05) and prevalence of GSTM1 polymorphism.

## 4. Discussion

There are a lot of epidemiological and genetical studies with the expectation to monitorize the risk of lung cancer using specific biomarkers. GST gene polymorphism is one of the subject of matter. A number of studies have been tried to establish the relationship between polymorphic expression of different GSTs and lung cancer risk in different ethnic populations [7,10,15,16], and the results have been conflicting [15,17]. One reason for the discrepancies could be the fact that most studies were conducted in different populations (one of the discrepancies that we encountered is due to holding the study in different populations). However, none of the main characteristics of the subjects explain satisfactorily the apparent discrepancies (i.e. race, histological type and level of smoking). Different histological subtypes of lung cancer, in particular may also be related to respective exposures or factors, and thus need to be analyzed separately [15,18].

The M1 variant of GST (GSTM1) detoxifies reactive intermediates of PAHs and other carcinogens. Although, the relationship between GSTM 1 polymorphism and lung cancer has been studied by various investigators, the effect of GSTM 0/0 null allele has not been explained clearly yet. A significant association of GSTM1 null genotype with lung cancer has already been observed in two large studies belongs to Japanese [19,20] and two in Chinese [21,22]. Furthermore, a study in Caucasians reported a significant association between lung adenocarcinoma and the GSTM1 null genotype [23]. In a meta-analysis study by Mc. Williams et al., it was shown that GSTM 0/0 null allele was a risk factor for the development of the lung cancer [24]. A meta-analysis of 11 studies found an OR of 1.6 (95% CI = 1.26–2.04) for an association between the GSTM1-null genotype and lung cancer risk [25]. A meta-analysis published by Simone Benhamou and co-workers reported that there was no statistically significant relationship between carrying GSTM null genotype and susceptibility to lung cancer but the number of the patients carrying this genotype was higher in the lung cancer group [12]. Although Pinarbasi et al. [26] reported a correlation between GTSM1 and lung cancer (p = 0.0001) in the Turkish population, the other trials conducted by Aras et al. [27] and Ozturk et al. [28] in Turkish population revealed the contrary (p > 0.05) (Table 4). In our study, we found no statistically significant relation between GSTM null genotype and susceptibility to lung cancer. Additionally, the rate of GSTM null genotype was higher in control group than cancer patients.

The frequencies of polymorphic genes in control populations have been reported to be different in various ethnic groups. In addition, intra-ethnic differences have been established [8,9]. GSTM1 frequencies range from 42 to 60% in Caucasians [8]. GSTM1 null genotype has been shown to be 31 to 66% in Asians, Indians and Caucasians [28-30]. On the other hand, GSTM1 deletion polymorphism for African-Americans was found to be 23–35% [31] and for Chileans was 21% [10]. In these series the frequency of GSTM1 null genotype was 52,6% which was similar to some European countries (Germany, Denmark, and France), Canada, and Korea (Table 4).

When the control groups of studies performed in Turkish population are reviewed, the frequency of GSTM1 null genotype was found to be 41.2% (n = 1809). This figure is lower than European countries, United States, Saudi Arabia, Japan, Singapore, and Korea (Table 4).

Several studies have also been carried out in this regard in Turkish populations. However, some need verification and others are contradictory. GSTM1 null genotype has been shown to be 18 to 66% in Turkish population (n = 1809) (Table 3). In Ozturk and co-workers study [28], GSTM1 null genotype incidence was found to be 49.2% in Turkish population. While Aktas et al. [27] found the prevalence of null polymorphism 34.7%; Pinarbasý et al. [26] detected it to be 18% in Turkish population. The reason for this difference between both studies was attributed to regional variation of the controls included in these studies by Pýnarbaşı. While Pýnarbaşı included only individuals from Central Anatolia region of Turkey, Aktas did not report such a restriction [26]. In the current series the frequency of GSTM1 genotype was found to be 52.6% and is the highest among the series in Turkish population except series of Aras. The possible explanation for the high rates of the current series and Aras' series could be the inclusion of subject living in two large cities of Turkey, Istanbul and Ankara containing people from all over the country. In our previous study, there was no significant relationship between lung cancer and gene polymorphism and we had concluded that insignificancy was due to subject number inadequacy [7] but as we have involved more subjects, we could not improve the significance.

In conclusion, we observed that carrying the GSTM1 genotype is not a risk factor for lung cancer, alone. The frequencies of GSTM1-null genotype in control Turkish populations have been observed to be intra-ethnic differences. In future, the risk of lung cancer is expected to be monitorized using specific biomarkers in genetic researches.

**Table 3:** The frequency of GSTM1 null genotype in Turkish population

Studies of Turkey	Genotypes	Patients (n =)	Percentage	Healthy Controls (n =)	Percentage	OR(95%) CI	p value
Pýnarbaşı et al (2003) (26)*	GSTM1 null	101	48%	206	18%	4.14(2.36–7.27)	p = 0.0001
Aras et all. (ANK) (27)*	GSTM1 null	54	72.7	100	66%	0.73(0.33–1.59)	p > 0.05
Öztürk et al. (2003) (28)*	GSTM1 null	55	47.3%	65	49.2%		p > 0.05
Curent series*	GSTM1 null	102	49%	154	52.6%	1.397(0.70–1.90)	p = 0.57
Ada et al. (2004) (9)	GSTM1 null	-	-	133	51.9%	-	-
Özbek et al. (2001)(32)	GSTM1 null	-	-	130	47%	-	-
Seyitoğlu et al. (2003) (33)	GSTM1 null	-	-	200	47%	-	-
Aktas et al. (2001) (34)	GSTM1 null	-	-	172	34.7%	-	-
Toruner et al. (2001) (35)	GSTM1 null	-	-	121	45.5%	-	-
Tamer et al. (2004) (36)	GSTM1 null	-	-	103	40.8%	-	-
Tursen et al. (2004) (37)	GSTM1 null	-	-	178	24.2%	-	-
Tamer et al. (2004) (38)	GSTM1 null	-	-	247	41.7%	-	-
*Studies of Turkey(Total)*	*GSTM1 null*	-	-	*1809*	*41.2%*	-	-

* Studies with lung cancer patients

**Table 4:** The frequency of GSTM1 null genotype in control populations, geographic distribution

Studies (year)	Country	Genotypes	Healthy controls (n =)	Percentage
Garte et al. (2001)	Denmark	GSTM1 null	537	53.6%
Garte et al. (2001)	Finland	GSTM1 null	482	46.9%
Garte et al. (2001)	France	GSTM1 null	1184	53.4%
Garte et al. (2001)	Germany	GSTM1 null	734	51.6%
Garte et al. (2001)	Italy	GSTM1 null	810	49.4%
Garte et al. (2001)	Spain	GSTM1 null	192	49.4%
Garte et al. (2001)	Sweden	GSTM1 null	544	55.9%
Garte et al. (2001)	United Kingdom	GSTM1 null	1112	57.8%
Garte et al. (2001)	Netherlands	GSTM1 null	419	50.4%
Garte et al. (2001)	Norway	GSTM1 null	423	50.6%
Garte et al. (2001)	Portugal	GSTM1 null	501	58.3%
Garte et al. (2001)	United States	GSTM1 null	1751	54.3%
Garte et al. (2001)	Saudi Arabia	GSTM1 null	895	56.3%
Garte et al. (2001)	Canada	GSTM1 null	304	51.3%
Garte et al. (2001)	Singapore	GSTM1 null	244	56.2%
Garte et al. (2001)	Korea	GSTM1 null	165	52.1%
*Studies of Turkey (see Table 3)*	*Turkey*	*GSTM1 null*	*1809*	*41.2%*

## Competing interests

The author(s) declare that they have no competing interests.
